# Maternity Service in Bristol

**Published:** 1952-04

**Authors:** A. G. Heron


Figures we are told can prove anything. Cannot we deduce from those quoted by Professor Lennon in his stimulating contribution to the January *Journal* that Bristol has been relatively successful in reducing maternal deaths to the occasional unavoidable ones from haemorrhage or toxaemia? I think also that there will be very mixed views concerning the Professor's ideas on domiciliary midwifery. If conditions are satisfactory there is much to be said in favour of confinement at home. If this is any less safe for the normal case it has only become so in quite recent years. In this area however not only are home conditions in a great many cases unsatisfactory with regard to accommodation and help, but for a long time there has been a conditioning towards hospitals or nursing homes.

The Minister has recognized this latter fact, and made no attempt to enforce domiciliary midwifery.

We realize that a certain number of normal cases are required for teaching purposes but it is surely entirely wrong that all " social grounds " cases should occupy specialist beds and especially at the appalling cost of £20 (approx.) weekly. That is one reason why general practitioners are pressing for smaller, simpler, units where they can look after these cases themselves, and why we have been striving to get support for efficient private maternity homes. These often represent the life savings of midwives who, with a true sense of vocation and real hard work, give satisfaction to all concerned, and manage to earn a livelihood at a charge of about 10 guineas weekly. It is unjust and stupid for these homes to be crushed by an unwieldy colossus, and a great pity that many young couples who would prefer otherwise, are compelled by circumstances to have their babies expensively mass-produced. But the Treasury has directed that the only way in which it could support such an economical unit would be by taking it over, thereby sounding the death knell of economy. We have already lost one excellent home accustomed to dealing with 500 cases a year. Must the others go too?

However, that is not the Professor's fault. I am very grateful for his interest in these " extra-mural " problems, his recognition that all is not well in Bristol, and his emphasis on the necessity for team-work and real co-operation.

A. GORDON HERON

